# Effect of ionic liquid treatment on the ultrastructural and topochemical features of compression wood in Japanese cedar (*Cryptomeria japonica*)

**DOI:** 10.1038/srep30147

**Published:** 2016-07-18

**Authors:** Toru Kanbayashi, Hisashi Miyafuji

**Affiliations:** 1 Department of Wood Improvement, Forestry and Forest Products Research Institute, Tsukuba, Japan; 2 Graduate School of Life and Environmental Sciences, Kyoto Prefectural University, Kyoto, Japan

## Abstract

The morphological and topochemical changes in wood tissues in compression wood of Japanese cedar (*Cryptomeria japonica*) upon treated with two types of ionic liquids, 1-ethyl-3-methylimidazolium chloride ([C2mim][Cl]) and 1-ethylpyridinium bromide ([EtPy][Br]) were investigated. Compression wood tracheids were swollen by both ionic liquids but their swelling behaviors were different in the types of ionic liquids used. Under the polarized light, we confirmed that crystalline cellulose in compression wood is amorphized by [C2mim][Cl] treatment whereas it changes slightly by [EtPy][Br] treatment. Raman microscopic analyses revealed that [C2mim][Cl] can preferentially liquefy polysaccharides in compression wood whereas [EtPy][Br] liquefy lignin. In addition, the interaction of compression wood with ionic liquids is different for the morphological regions. These results will assist in the use of ionic liquid treatment of woody biomass to produce valuable chemicals, bio-fuels, bio-based composites and other products.

The utilization of renewable lignocellulosics for alternative to fossil resources has attracted great interest to solve the world-wide problems of global warming and future shortage of energy-generating resources. Above all, woody biomass has been considered to be one of the most expected natural resource due to its prominent properties, such as carbon neutral, massive stockpile, and non-competitiveness with food resources. There have been extensive efforts to mitigate its high recalcitrant nature and develop efficient conversion and utilization methods^1^. However, such technologies have not been established; thus, further improvement is necessary.

Ionic liquid is a general term for organic salts with a low melting point around or below 100 °C. It has many unique properties: negligible vapor pressure, chemical and thermal stability, non-flammability, low viscosity, and recyclability^2^. In addition, certain ionic liquids have ability to liquefy cellulose^3^,^4^,^5^ and even wood^6^,^7^,^8^. In recent years, ionic liquid has been gained great deal of attention as a novel environmental-friendly solvent for converting woody biomass into valuable chemicals, bio-fuels, and biomaterials^9^. To develop effective conversion technology by means of ionic liquid treatment, many fundamental investigations with respect to the interaction between wood and ionic liquids have been carried out from various viewpoints, including chemistry^10^,^11^,^12^,^13^, topochemistry^14^,^15^,^16^,^17^,^18^, and morphology^19^,^20^,^21^,^22^,^23^.

In general, when the coniferous trees grow under the stress such as wind and gravitational force, abnormal wood parts which are called compression wood occur on the lower side of leaning stems and branches^24^. The anatomical structure and chemical composition of compression wood are distinctively different from those of normal wood. Compression wood forms thick circular outlined tracheids, intercellular spaces at the cell corner and helical cavity on inner surface of secondary wall, and is absent from inner layer of secondary wall^25^. From the chemical point of view, compression wood has higher lignin and galactan content but lower cellulose and galactoglucomannan content than normal wood^26^. In addition, lignin in compression wood has large proportion of *p*-hydroxyphenyl propane units^27^. From these properties, compression wood is generally difficult to use for industrial processing^28^. However, softwood especially juvenile wood is estimated to contain non-negligible amounts of compression wood^29^. In addition, compression wood is formed even in perfectly straight and vertical trees^30^. With increasing in global wood demands, short-rotation afforestation and complete-tree utilization has been emphasized. The development of efficient conversion process of compression wood makes it possible to use woody biomass effectively without waste.

Although a number of studies on ionic liquid treatment of wood have already been performed, the knowledge of the impact of ionic liquid treatment on compression wood is very little^31^ especially in morphological point of view. Thus, in the present work, we conducted ultrastructural and topochemical characterization of compression wood cell walls treated with ionic liquids by means of light microscopy, scanning electron microscopy (SEM), and confocal Raman microscopy. 1-Ethyl-3-methylimidazolium chloride ([C2mim][Cl]) and 1-ethypyridinium bromide ([EtPy][Br]) were selected as the ionic liquid used which prefer to react with cellulose^10^ and lignin^13^, respectively. We showed the differences in interaction of compression wood with ionic liquid among the morphological regions of cell walls and the types of ionic liquids.

## Results and Discussion

### Light microscopy analysis

**Figure 1:**
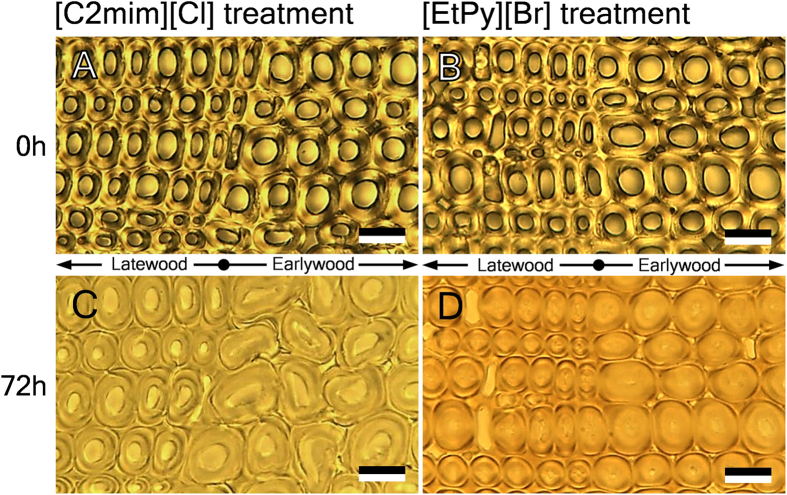
Bright-field images of transverse sections in compression wood before (**A**,**B**) and after treatment with [C2mim][Cl] (**C**) and [EtPy][Br] (**D**) at 120 °C for 72 h. Scale bars: 20 μm.

The morphological changes in compression wood tissues during ionic liquid treatment were observed by bright-field microscopy (Fig. 1). Compression wood tracheids in earlywood were collapsed after [C2mim][Cl] treatment, but not in latewood (Fig. 1C). This result was contrary to the changes in normal wood. It is reported that treatment with [C2mim][Cl] lead to distortion and dissociation of normal wood tracheids in latewood, but not in earlywood^21^. With regard to the compression wood tracheids treated with [EtPy][Br], no significant morphological changes were observed in both earlywood and latewood (Fig. 1D) as with the result of normal wood^16^.

**Figure 2:**
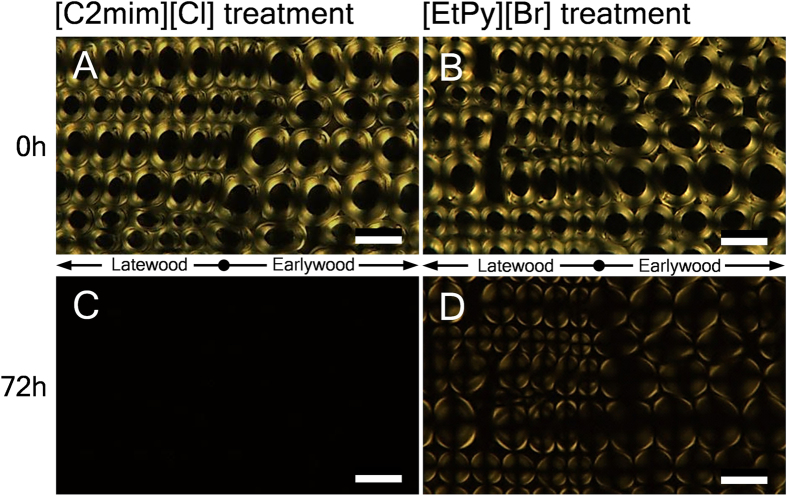
Polarized light microscopy images of transverse sections in compression wood before (**A**,**B**) and after treatment with [C2mim][Cl] (**C**) and [EtPy][Br] (**D**) at 120 °C for 72 h. Scale bars: 20 μm.

We performed polarized light microscopy observation at the same positions that were observed by bright-field microscopy (Fig. 2). The brightness from the birefringence of crystalline cellulose could be clearly seen in untreated samples (Fig. 2A,B). After 72 h of [C2mim][Cl] treatment, the brightness disappeared completely (Fig. 2C); nevertheless the cell walls preserved their forms as seen in bright-field images (Fig. 1C). Meanwhile, after 72 h of [EtPy][Br] treatment, the brightness has been still visible even though the brightness decreased slightly (Fig. 2D). These results imply that the crystalline structure of cellulose in compression wood is broken by [C2mim][Cl] treatment before complete liquefaction of the cell walls, but is barely affected by [EtPy][Br] treatment.

**Figure 3:**
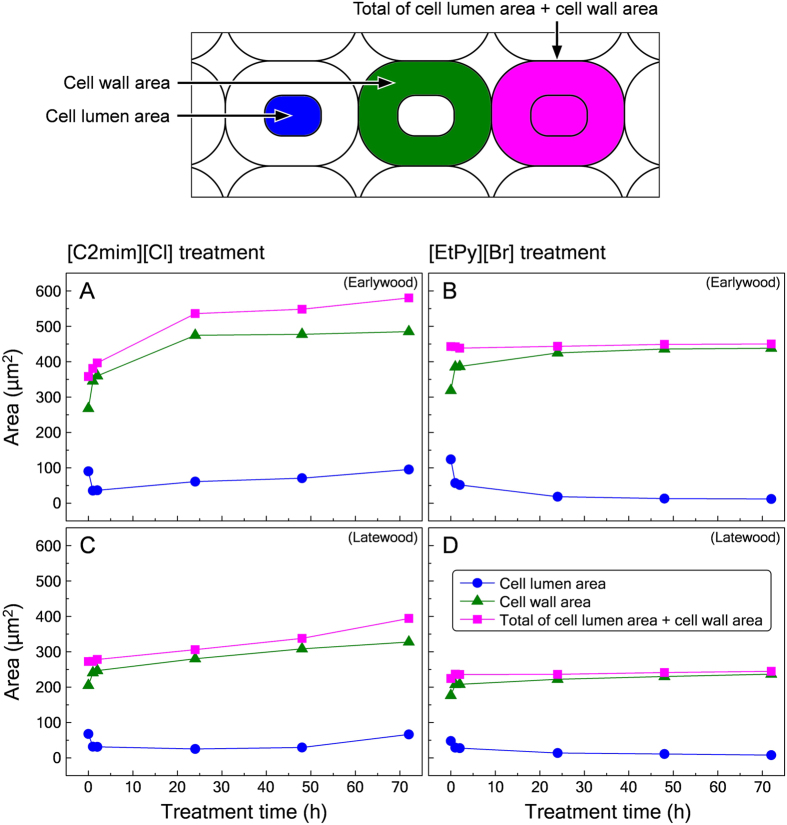
Changes in cell lumen area, cell wall area and total of cell lumen area + cell wall area for compression wood tracheids in earlywood (**A**,**B**) and latewood (**C**,**D**) during [C2mim][Cl] (**A**,**C**) and [EtPy][Br] (**B**,**D**) treatment. In upper panel, definition of cell lumen area, cell wall area and total of cell lumen area + cell wall area are presented. These three areas were measured for specific five tracheids and the average was calculated using image analysis software (Motic Image Plus 2.2S).

Swelling process of compression wood tracheids during ionic liquid treatment was determined by means of measuring three areas: cell lumen area, cell wall area, and total of cell lumen and cell wall area; the results were described in Fig. 3. All the tracheids showed inward swelling in an early stage of both ionic liquids treatment. In the case of [C2mim][Cl] treatment, the total of cell lumen and cell wall area in earlywood increased significantly after 24 h (Fig. 3A), while that in latewood increased just a little (Fig. 3C). In addition, at 72 h of treatment, the cell wall areas in earlywood and latewood had increased by 1.8 and 1.6 times, respectively. On the other hand, after 72 h of [EtPy][Br] treatment, the cell wall areas in both earlywood and latewood had increased by only 1.3 times without outward swelling (Fig. 3B,D). From the above results, the collapse of earlywood tracheids during [C2mim][Cl] treatment as shown in Fig. 1C can be considered to arise from the significant outward swelling of cell walls.

Previously, we reported that the cell wall areas in latewood of normal wood tracheids increased by 5 times after 48 h of [C2mim][Cl] treatment^21^. In addition, in the case of [EtPy][Br] treatment, the cell wall areas in earlywood and latewood of normal wood tracheids increased by 1.3 and 2 times after 72 h, respectively^16^. Thus, the degree of swelling for compression wood tracheids was gentler than normal wood tracheids. These differences were mainly caused by the ultrastructure and chemical composition of tracheids. As the one reason, there is difference in microfibril angle. In general, swelling of the cell walls is mainly attributed to swelling of the bundles of cellulose microfibrils for perpendicular direction to the fibril axis^32^. The more the angle between microfibrils and longitudinal cell axis decreases, the more the swelling ratio toward perpendicular to longitudinal cell axis increases. The microfibril angle in middle layer of secondary wall (S_2_) which composes large part of the cell wall in compression wood tracheids are higher than in normal wood tracheids; their microfibril angles are considered to be approximately 5–20°^33^ and 45°^34^, respectively. In addition, the inner layer of secondary wall (S_1_) which has very high microfibril angle (approximately 70–90°) in compression wood tracheids is considerably thicker than in normal wood^24^. Thus, compression wood tracheids are difficult to swell outwards rather than normal wood tracheids. As another reason, there is difference in chemical composition. It is reported that compression wood tracheids contain more lignin and less cellulose than normal wood tracheids^26^. Our previous study revealed that lignin has high recalcitrance to [C2mim][Cl] attack compared with cellulose and hemicellulose^10^. Thus, the reactivity of [C2mim][Cl] with compression wood tracheids were lower than with normal wood tracheids. From the above factors, the high microfibril angle of S_2_, high proportion of S_1_ and high lignin concentration, caused gentle swelling of compression wood tracheids during ionic liquid treatment.

### SEM observation

**Figure 4:**
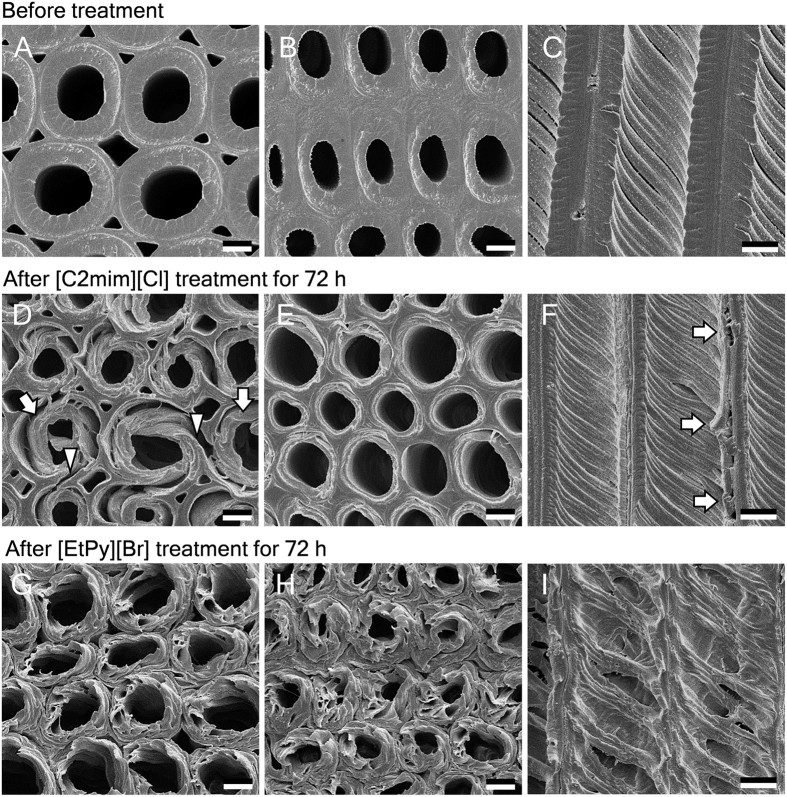
SEM images of compression wood tracheids before (**A**–**C**) and after treatment with [C2mim][Cl] (**D**–**F**) and [EtPy][Br] (**G**–**I**) at 120 °C for 72 h. Left, transverse views of earlywood; middle, transverse views of latewood; right, radial views of earlywood. Arrows, dissociated S_2_; arrowheads, ruptured S_1_ and CML. Scale bars: 5 μm.

We analyzed transverse and radial surface of intact and ionic liquid treated samples by SEM (Fig. 4). As can be seen from the transverse sections of tracheids treated with [C2mim][Cl], S_2_ was dissociated from S_1_ (indicated by arrows) and S_1_ and compound middle lamella (middle lamella + primary wall; CML) were partially torn (indicated by arrowheads) in earlywood (Fig. 4D). These breakage and dissociation were caused by the significant swelling of S_2_ (as seen in Fig. 1C) and decreasing in adhesion between S_1_ and S_2_ during the treatment. With respect to latewood, no significant destruction occurred but the thickness of the cell walls was decreased due to the liquefaction of chemical components of the cell walls (Fig. 4E). These morphological changes could also be seen in radial section (Fig. 4F). On the other hand, after treatment with [EtPy][Br], many pores were formed on the transverse and radial surface of the cell walls in both earlywood and latewood (Fig. 4G–I). Previously, we have also observed them on normal wood tracheids^16^ and wood fibers^17^ treated with [EtPy][Br]. In addition, the other treatments such as steam explosion^35^, ammonia fiber expansion^36^ and alkaline treatment^37^ have been reported to result in the pore formation. Therefore, the pores thought to be formed by delignification. The formation of porous structure by means of [EtPy][Br] treatment will improve accessibility of enzyme followed by development of effective bio-conversion technology such as enzymatic saccharification.

### Confocal Raman microscopy analysis

To understand the chemical changes in compression wood tracheids during ionic liquid treatment at the cellular level, chemical mapping was performed to visualize the distribution of wood components using Raman mapping technique. It is reported that the anatomy and chemical composition of the compression wood tissues other than tracheids are similar to normal wood^24^. Thus, Raman analysis was done on only tracheids. In this study, cellulose and hemicellulose were collectively called polysaccharides because they are difficult to identify each other in the Raman spectrum of wood^38^,^39^. The band positions around 1,600 and 2,900 cm^−1^ have been assigned to aromatic ring vibration of lignin and C–H and C–H_2_ stretching of polysaccharides, respectively^40^. The distribution of lignin and polysaccharides were constructed using the band regions 1,585–1,606 cm^−1^ and 2,876–2,902 cm^−1^, respectively.

**Figure 5:**
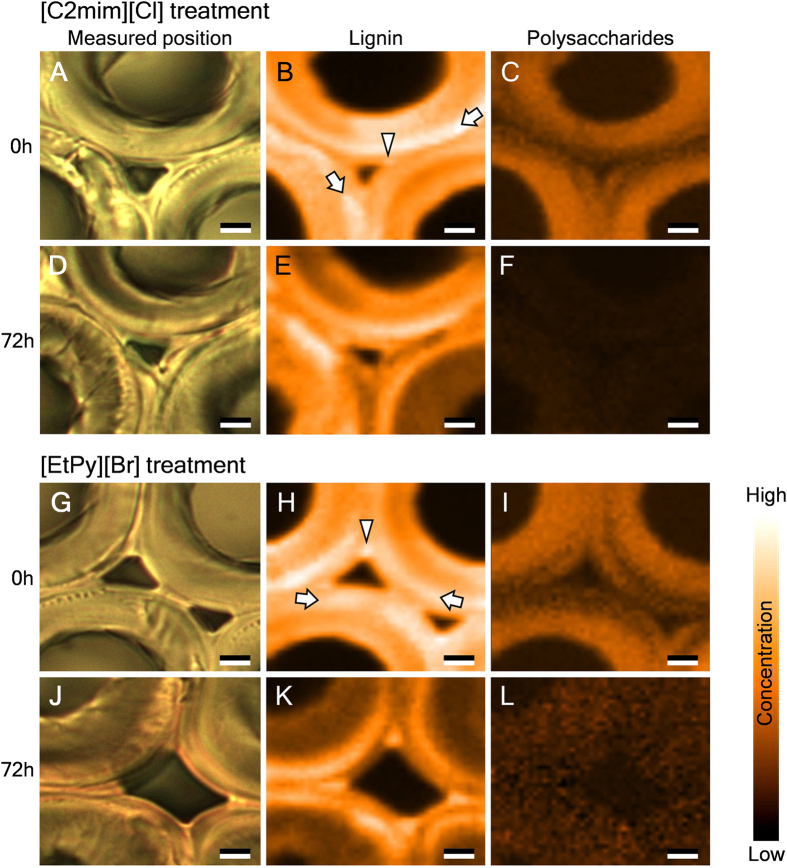
Raman mapping analysis on transverse sections of compression wood tracheids before and after treatment with [C2mim][Cl] (**A**–**F**) and [EtPy][Br] (**G**–**L**) at 120 °C for 72 h. Left, bright-field images of the measured position; middle, distribution of lignin (1,585–1,606 cm^−1^); right, distribution of polysaccharides (2,876–2,902 cm^−1^). Bright regions represent high concentrations of specific chemical compositions, whereas dark regions represent low concentrations. Arrows, S_2_(L); arrowheads, CML. Scale bars: 3 μm.

Results from the Raman mapping performed on the compression wood tracheids before and after ionic liquid treatment were described in Fig. 5. In the Raman images, the bright areas represent high concentration of specific chemical composition, whereas the dark areas represent low concentration. Before the treatment, outer S_2_ layer [S_2_(L)] (indicated by arrows) and CML (indicated by arrowheads) were highly lignified as with the previous reports^41^. The lignin concentration changed slightly after [C2mim][Cl] treatment (Fig. 5E), but decreased significantly after [EtPy][Br] treatment except for the areas such as S_2_(L) and CML (Fig. 5K). Differences in molecular structure and concentration of lignin and penetrability of ionic liquid may cause the differences in the reactivity of ionic liquids among the morphological regions. Meanwhile, the concentration of polysaccharides decreased significantly and its signal was almost disappeared after [C2mim][Cl] treatment (Fig. 5F), but still have been detected after [EtPy][Br] treatment (Fig. 5L). These results indicate that polysaccharides in compression wood tracheids readily reacts with [C2mim][Cl] while lignin reacts with [EtPy][Br]. In addition, lignin in S_2_(L) and CML has high recalcitrance to reactions with ionic liquids than other morphological regions.

## Material and Methods

### Samples and Chemicals

Compression wood samples were collected from the branch of Japanese cedar (*Cryptomeria japonica*). These samples were cut in to small blocks (approximately 5 × 5 × 5 mm^3^) that were extracted with ethanol/benzene (1:2, v/v) for 24 h in a Soxhlet apparatus. The extracted samples were dried for 24 h in an oven at 105 °C prior to further treatment. The ionic liquids, [C2mim][Cl] and [EtPy][Br], were purchased from Tokyo Chemical Industry Co., Ltd. (Tokyo, Japan).

### Light microscopy analysis

The extracted samples were sectioned to 15-μm-thick with a sliding microtome (TU-213, Yamato Kohki Industrial Co., Ltd., Saitama, Japan) and mounted in a 20-μm-deep hemocytometer (Sunlead Glass Corp., Saitama, Japan). The mounted sections were dried for 2 h at 105 °C before adding 100 μL of ionic liquid that was heated to 120 °C by dropping the ionic liquid onto the mounted section. The hemocytometer was closed with a glass cover after adding the ionic liquid. Then it was placed in an oven at 120 °C for various time periods. After a specified treatment time, the hemocytometer was analyzed using light microscopy (BH-2, Olympus, Tokyo, Japan) to examine morphological changes in the wood section. Three areas (cell lumen area, cell wall area, and total of cell lumen area + cell wall area; defined as illustrated in Fig. 3) were measured for specific five cells, using image analysis software (Motic Image Plus 2.2S, Shimadzu Rika Corporation, Tokyo, Japan) and the average was calculated.

### SEM observation

The extracted samples were surfaced with a sliding microtome. The surfaced samples were dried for 24 h at 105 °C and the surfaced area was treated by dipping into ionic liquid and heating to 120 °C for various periods of time. During dipping treatment, the ionic liquid was stirred gently with a magnetic stirrer. The treated specimens were dipped in dimethylsulfoxide (DMSO) to remove ionic liquid and then washed with distilled water to remove DMSO. After drying for 24 h at 105 °C, each specimen was mounted on a specimen holder using carbon tape and sputter-coated with Au using a JEOL JFC-1600 auto fine coater (Tokyo, Japan). The exposed surface was examined by SEM (JSM-5510LV, JEOL) at an accelerating voltage of 10 kV.

### Confocal Raman microscopy analysis

For Raman microscopy, the ionic liquid treatment was the same as that for light microscopy. To perform analysis on the same cell wall area, 15-μm-thick serial sections were prepared. After a specified treatment time, a large amount of distilled water was poured into the hemocytometer from the lateral direction, and then the hemocytometer was placed in a Petri dish filled with distilled water for 24 h at room temperature to remove ionic liquid completely. The samples were analyzed by a confocal microRaman system (LabRAM ARAMIS, Horiba Jobin Yvon, Longjumeau, France) equipped with a confocal microscope (BX41, Olympus) and a motorized x, y stage. To obtain high spatial resolution, the measurements were performed with an oil immersion objective having a high numerical aperture (NA) (NA = 1.40, UPLSAPO 100XO, Olympus). The instrument was equipped with a diode-pumped solid-state laser (λ = 532 nm, Ventus VIS 532, Laser Quantum, Cheshire, UK). The incident laser power on the sample was approximately 10 mW. The scattered Raman light was detected by a CCD detector behind a 300 lines/mm grating. The confocal pinhole diameter was 300 μm. The data acquisition and analysis were done by means of LabSpec5 software (Horiba Jobin Yvon). The measurements were conducted every 0.4 μm and the spectra were obtained by averaging 4 cycles, each of 0.1 s integration time. To remove background from fluorescence, the raw spectral data were baseline corrected.

## Conclusions

We investigated the interaction of compression wood cell walls with ionic liquids from a morphological and topochemical point of view. Both [C2mim][Cl] and [EtPy][Br] treatments induced swelling of compression wood cell walls, but their swelling behaviors were gentler than normal wood. The crystalline cellulose was amorphized by [C2mim][Cl] treatment while it was slightly changed by [EtPy][Br] treatment. Although lignin was easy to react with [EtPy][Br], lignin in S_2_(L) and CML was less reactive. Consequently, the interaction of ionic liquids with compression wood cell walls was different for the types of ionic liquids and the morphological regions at the cellular level.

## Additional Information

**How to cite this article**: Kanbayashi, T. and Miyafuji, H. Effect of ionic liquid treatment on the ultrastructural and topochemical features of compression wood in Japanese cedar (*Cryptomeria japonica*). *Sci. Rep.* **6**, 30147; doi: 10.1038/srep30147 (2016).
